# Supplementary material for: Effects of Dietary Supplementation with Aurantiochytrium sp. on Zebrafish Growth as Determined by Transcriptomics
Source: Animals (Basel). 2022 Oct 16;12(20):2794. doi: 10.3390/ani12202794 (PMC9597791; doi:10.3390/ani12202794)
Supplement: Supplementary file 1 [file animals-12-02794-s001.zip › animals-1926007-supplementary.pdf]

Table S1 *Aurantiochytrium* Extract Main Ingredients

Major fatty acids	Content
Methyl tetradecanoate	2.34%
Pentadecanoic acid, methyl ester	2.44%
Hexadecanoic acid, methyl ester	30.21%
Methyl4, 7, 10, 13, 16-docosapentaenoate	9.07%
4, 7, 10, 13, 16, 19-docosahexaenoic acid, methylester	50.19%

Table S2 The Product Numbers of Commercial Assay kits.

**Enzyme**	**Kit No.**	**Manufacturer**
Amylase (AMS)	C016-1-1, C016-2-1	Nanjing Jiancheng Bioengineering Institute
Glucose-6-phosphate dehydrogenase (G-6-PD)	A027-1-1	Nanjing Jiancheng Bioengineering Institute
Malic enzyme (ME)	BC1125	Solarbio Life Sciences
pyruvate kinase (PK)	A076-1-1	Nanjing Jiancheng Bioengineering Institute
Acyl-CoA oxidase (ACO)	H232	Nanjing Jiancheng Bioengineering Institute
Lipase (LPS)	E1019	Applygen Technologies Inc.
Fatty acid synthase (FAS)	H231	Nanjing Jiancheng Bioengineering Institute
Carnitine-acylcarnitine translocase (CACT)	SEB657Ra	Wuhan Cloud-Clone Corp.
Acetyl-CoA carboxylase (ACC)	H232	Nanjing Jiancheng Bioengineering Institute
Superoxide dismutase (SOD)	A001-3-1	Nanjing Jiancheng Bioengineering Institute
Phospholipid hydroperoxide glutathione peroxidase (GSH-PX)	A005-1-1	Nanjing Jiancheng Bioengineering Institute
Catalase (CAT)	A007-1-1	Nanjing Jiancheng Bioengineering Institute
malondialdehyde (MDA)	A003-1-1	Nanjing Jiancheng Bioengineering Institute

Table S3 Primers Used for Detection of DEGs

Gene	Primer name	Primer sequence (5′ -3′)	Purpose	Accession No.
Odc1	*Odc1*-F	TCAGACTTTGACTTCGCCTTCC	RT-qPCR	ENSDARG00000007377
	Odc1-R	TGACCACCGCCCTGCTAT	RT-qPCR	
mgst1.1	*mgst1.1*-F	GATGTGGAAAGAGTGCGACGAT	RT-qPCR	ENSDARG00000032618
	mgst1.1-R	TGGTAGGTTGCGGAAGAGCC	RT-qPCR	
gsto2	*gsto2*-F	AGTGCCAGTGTTGGAAACCT	RT-qPCR	ENSDARG00000033285
	gsto2-R	CTCTCAAACGGGTCAGACGG	RT-qPCR	
gpx4a	*gpx4a*-F	GGCAGTCATGCGTTTCTTAGG	RT-qPCR	ENSDARG00000068478
	gpx4a-R	TCTCAGAGTACTTGGCGTGC	RT-qPCR	
rrm2	*rrm2*-F	TTCGCTTCAGCTGGACCGTAG	RT-qPCR	ENSDARG00000078069
	rrm2R	TGGGTGGCGTGTTTTCTTTGT	RT-qPCR	
oga	*oga*-F	GGAGATGTGCTGCTCTGTCA	RT-qPCR	ENSDARG00000074686
	oga-R	ACGACACCCTAACCACTGAAC	RT-qPCR	
ppp1r3cb	*ppp1r3cb*-F	CGCAGCAAACCACTGTTCTT	RT-qPCR	ENSDARG00000014554
	ppp1r3cb-R	GCAGAACCCTGGTGCAATTC	RT-qPCR	
ugp2a	*ugp2a*-F	TAGTCGATCTGAGGCTCCCA	RT-qPCR	ENSDARG00000005578
	ugp2a-R	TCCTGAAACTCCGCCATTCC	RT-qPCR	
enpp1	*enpp1*-F	TGTTCATCTGCCGTTTGGGA	RT-qPCR	ENSDARG00000005789
	enpp1-R	CAGGAGGCCGAAGCTCATAG	RT-qPCR	
cyp46a1.2	*cyp46a1.2*-F	TGATAGCAGTTAGAGGGTAGTGT	RT-qPCR	ENSDARG00000004262
	cyp46a1.2-R	GTCGTCGGAGTACATCGCTT	RT-qPCR	
acsl1b	*acsl1b*-F	ACGAGTTCGGTCGAGTTCAG	RT-qPCR	ENSDARG00000003854
	acsl1b-R	GTGGCGTACCAGTATGCAGT	RT-qPCR	
ugt1b5	*ugt1b5*F	CTCACGAGCTGAACTGGCTT	RT-qPCR	ENSDARG00000089507
	ugt1b5-R	CTGGCTCAGTTGTCAGGCGT	RT-qPCR	
ugt1b4	*ugt1b4*-F	TGTCCGAACCCACCCTCTTA	RT-qPCR	ENSDARG00000097024
	ugt1b4-R	ACCCAACATAAGCCTACACATCA	RT-qPCR	
ugt1b3	*ugt1b3*-F	GGGTCCAGGCAAACACTACA	RT-qPCR	ENSDARG00000097979
	ugt1b3-R	AGTCACAGTCTTCAAACGGG	RT-qPCR	
lpcat4	*lpcat4*-F	CCGGAATCCAGGAAGAAGTGC	RT-qPCR	ENSDARG00000035028
	lpcat4-R	ACAGTCCTTTCCAGGTCCAG	RT-qPCR	
dhrs13l1	*dhrs13l1*-F	GGGCACCATTGACTTCGACT	RT-qPCR	ENSDARG00000098746
	dhrs13l1-R	GCAGGTCACATTGGTTCCCT	RT-qPCR	
nek2	*nek2*-F	GGACTATGGCACTATGGCCG	RT-qPCR	ENSDARG00000005619
	nek2-R	TTCGGTCATGGTATCGGACG	RT-qPCR	
ctps1b	*ctps1b*-F	TCGGAACGGAGCAGTTTCAG	RT-qPCR	ENSDARG00000098386
	ctps1b-R	GCTGGCGATGATGCCTTTAC	RT-qPCR	
β-actin	*β-actin*-F	GGCATCACACCTTCTACAA	Reference gene	NM_131031.2

β-*actin*-R	CAGAGTCCATCACAATACCA	Reference gene
